# Natural bioactive compounds targeting the macrophage clock in asthma: evidence, challenges, and perspectives

**DOI:** 10.3389/fimmu.2026.1922510

**Published:** 2026-08-21

**Authors:** Baoyi Ni, Ru Chen, Shaobin Sun, Zhu Liu, Xi Zhang, Huan He, Jia Wang

**Affiliations:** 1 Mudanjiang Medical University, Mudanjiang, China; 2 Hongqi Hospital Affiliated to Mudanjiang Medical University, Mudanjiang, China; 3 Xuyi County People ‘s Hospital, Huai’ an, Jiangsu, China

**Keywords:** airway remodeling, asthma chronotherapy, circadian clock, natural chronobiotics, pulmonary macrophages

## Abstract

**Background:**

Nocturnal asthma and airway remodeling are intimately linked to pulmonary circadian dysfunction. The dysregulation of core clock genes (e.g., Bmal1, Rev-erbα) in macrophages drives metabolic reprogramming and pro-fibrotic signaling. Pharmacological modulation of this clock using natural bioactive compounds offers a promising therapeutic strategy.

**Purpose & scope:**

This review critically evaluates the *in vivo* evidence of natural chronobiotics (including resveratrol, quercetin, curcumin, honokiol, and nobiletin) in reshaping the asthmatic microenvironment, specifically focusing on subepithelial fibrosis and airway smooth muscle hyperplasia. We then propose a validation framework to distinguish genuine clock-dependent effects from pleiotropic pharmacological noise.

**Key challenges:**

We identify two major translational bottlenecks. First, the “pleiotropy dilemma”: most existing *in vivo* studies fail to distinguish true clock-dependent modulation from clock-independent antioxidant and anti-inflammatory effects. Second, and more critically, the core clock target BMAL1 itself exhibits a fundamental dichotomy in allergic asthma—myeloid BMAL1 deficiency exacerbates acute eosinophilic inflammation but paradoxically suppresses chronic airway remodeling in a context-dependent manner. This underscores that correlative observations without genetic validation are insufficient, and that chronobiotic efficacy must be rigorously dissected across diverse asthmatic phenotypes.

**Future perspectives:**

To advance respiratory chronopharmacology, we propose a rigorous validation framework requiring macrophage-specific conditional knockout models (e.g., LysM-Cre; Bmal1 fl/fl). Finally, we highlight the integration of CD206-targeted inhalable nanocarriers, precision chronopharmacological dosing, and spatial transcriptomics as the definitive roadmap to translate natural chronobiotics into disease-modifying therapies for asthma.

## 1 Introduction

Asthma exhibits distinct circadian rhythmicity. Nocturnal asthma is defined by exacerbations of lower respiratory tract symptoms and declines in lung function occurring during sleep or early morning. Epidemiological data indicate that up to 75% of patients experience nocturnal exacerbations independent of sleep or environmental behavioral cycles (1). Crucially, the frequent onset of these nocturnal symptoms is not merely a marker of poor acute control; chronic nocturnal airway hyperresponsiveness inflicts persistent mechanical stress and localized inflammation, ultimately driving irreversible airway remodeling. Clinical studies demonstrate that pulmonary function reaches its nadir during the biological night, a decrease that correlates with diurnal variations in airway mechanics, airway hyperresponsiveness, and airflow limitation (1, 2). Inhaled corticosteroids (ICS) remain the standard anti-inflammatory treatment for asthma; however, they primarily target downstream inflammatory mediators rather than directly regulating core circadian clock proteins, such as BMAL1 and REV-ERBα, which govern airway hyperresponsiveness (3, 4). Furthermore, inflammatory signals induce the rapid degradation of these clock proteins, a process not inhibited by ICS therapy. Consequently, anti-inflammatory treatment alone is often insufficient, limiting the control of nocturnal symptoms and necessitating additional bronchodilator use (5, 6).

As the first line of defense and central sentinels in the airway microenvironment, pulmonary macrophages are central to airway remodeling. They not only phagocytose allergens but also directly mediate fibroblast activation and airway smooth muscle hyperplasia through the secretion of pro-fibrotic factors (e.g., TGF-β1) and chemokines. Consequently, they serve as a critical hub connecting acute inflammation to chronic structural remodeling. The intrinsic molecular clock within these pulmonary macrophages regulates these local immune responses and immunometabolism. The transcription factor BMAL1 functions as a primary metabolic regulator. Decreased BMAL1 expression in myeloid cells shifts cellular metabolism toward glycolysis, driving succinate accumulation (7), which subsequently stabilizes hypoxia-inducible factor-1α (HIF-1α), activates the NLRP3 inflammasome, and increases IL-1β secretion (8). Concurrently, the circadian repressor REV-ERBα suppresses the transcription of pro-inflammatory genes (e.g., Il6, Ccl2) (9, 10). Reduced REV-ERBα function removes this suppression, promoting macrophage infiltration and persistent localized inflammation, which disrupts adjacent structural cells and drives irreversible airway remodeling (11, 12).

To intercept this circadian-driven pathology, pharmacological modulation of the molecular clock using natural bioactive compounds has emerged as a promising strategy. Unlike crude botanical extracts that exhibit chemical variability (13, 14), isolated natural bioactive compounds (e.g., polymethoxyflavones, polyphenols, and curcuminoids) offer defined structure-activity relationships. High-throughput screening and *in vitro* assays have identified specific compounds capable of modulating circadian targets, such as amplifying Bmal1 transcription or stabilizing clock proteins against stress-induced degradation (15, 16).

However, a critical translational gap exists in the current literature. Many of these identified natural chronobiotics are highly pleiotropic. In *in vivo* asthmatic models, it remains intensely debated whether their therapeutic efficacy—such as the attenuation of airway remodeling—stems from true clock-dependent modulation (chronobiology) or clock-independent, canonical anti-inflammatory and antioxidant pathways (e.g., direct ROS scavenging or NF-κB inhibition). Many existing studies rely on correlative observations rather than definitive mechanistic validation, making it challenging to differentiate specific chronobiotic effects from general pharmacological noise.

Therefore, this review aims to critically evaluate the current pharmacological evidence regarding natural bioactive compounds targeting the macrophage molecular clock in asthma. We will distinguish between *in vitro* mechanisms and asthma-specific *in vivo* evidence, confront the challenges posed by pleiotropic effects, and propose a rigorous validation framework—incorporating macrophage-specific conditional knockout models—to advance true chrono-pharmacotherapy in respiratory diseases.

## 2 Circadian rhythm dysfunction and macrophage polarization

### 2.1 The intrinsic molecular clock network in pulmonary macrophages

Pulmonary macrophages possess an autonomous molecular clock regulated by a transcription-translation feedback loop (TTFL) (17, 18). In macrophages, this molecular clock coordinates dual functions: it regulates immune responses primarily through the transcriptional repression of inflammatory cytokines, and it governs metabolic functions by modulating mitochondrial oxidative phosphorylation and glycolysis. Within this system, the transcription factors CLOCK (or its paralog NPAS2) and BMAL1 form a heterodimer that binds to E-box elements in the promoters of the Period (Per1/2/3) and Cryptochrome (Cry1/2) genes (18). The repression phase operates via dual mechanisms: the CRY-PER-casein kinase 1 (CK1) complex initially displaces CLOCK-BMAL1 from target promoters, after which CRY1 independently inhibits CLOCK-BMAL1 activity by blocking the recruitment of transcriptional coactivators (18). The 24-hour periodicity of this cycle is maintained through coordinated rhythmic phosphorylation and ubiquitin-proteasome degradation (18, 19).

An auxiliary stabilization loop consists of the opposing nuclear receptors REV-ERBα/β and RORα/γ, which compete for ROR response elements (ROREs) within the Bmal1 promoter (20, 21). While RORs activate Bmal1 transcription, REV-ERBs recruit the HDAC3 corepressor complex to repress it (21). REV-ERBα exerts its regulatory effects through dual genomic mechanisms. It directly binds ROREs to control core clock components, whereas for metabolic and immune regulation, cell-specific transcription factors such as PU.1 tether REV-ERBα to specific chromatin sites (21), enabling the repression of Il6 and Ccl2 transcription (9, 22).

Macrophage circadian rhythms are significantly governed by post-transcriptional mechanisms, with only approximately 15% of the circadian proteome corresponding to oscillating mRNA (23). This post-transcriptional regulation permits rapid immunometabolic adaptations; for instance, macrophage ATP production peaks during the active phase, whereas phagocytic capacity fluctuates independently of transcriptional alterations (23). Furthermore, alveolar macrophages (AMs) and interstitial macrophages (IMs) exhibit distinct epigenetic landscapes characterized by specific open chromatin patterns and lineage-determining factors (24, 25). The circadian rhythmicity of these tissue-resident macrophage populations is influenced by the local microenvironment. In precision-cut lung slices derived from allergic asthma models, macrophages display altered BMAL1 protein expression, reduced circadian amplitude, and phase shifts in core clock components (26).

### 2.2 BMAL1 deficiency and metabolic reprogramming

BMAL1 functions as a primary metabolic checkpoint in macrophages. Its deficiency induces a metabolic shift characterized by accelerated glycolytic flux and impaired Krebs cycle, leading to succinate accumulation (7, 27). Elevated intracellular succinate stabilizes hypoxia-inducible factor-1α (HIF-1α) and drives mitochondrial reactive oxygen species (ROS) production, which synergistically upregulate the transcription of pro-inflammatory genes such as Il1b (28–33). Furthermore, BMAL1 deletion directly compromises the NRF2-mediated cellular antioxidant defense system (34, 35).

These metabolic derangements culminate in NLRP3 inflammasome activation. BMAL1 deficiency abolishes the physiological time-of-day variation in mitochondrial membrane potential (8). The combination of elevated mitochondrial membrane potential and extracellular succinate engaging the SUCNR1 receptor promotes constitutive NLRP3 activation (8, 36). Concurrently, BMAL1 deficiency downregulates REV-ERBα expression, permitting C/EBPβ to bind the Saa1 promoter. This binding increases serum amyloid A1 production, inducing noncanonical inflammasome-mediated pyroptosis (37).

Crucially, while the aforementioned metabolic reprogramming pathways are primarily mapped in generalized inflammatory models (e.g., LPS stimulation), emerging evidence directly links these mechanisms to the asthmatic microenvironment. Clinical analyses reveal significant BMAL1 downregulation and circadian disruption in leukocytes and monocyte-derived macrophages of asthmatic patients (38). Consistently, in murine models of allergic asthma induced by house dust mite (HDM) or ovalbumin (OVA), pulmonary BMAL1 expression is markedly suppressed.

However, the exact net functional outcome of myeloid BMAL1 in asthma remains highly context-dependent, highlighting a critical area of conflicting findings in the current literature. For instance, Zasłona et al. demonstrated that myeloid-specific BMAL1 deletion (LysM-Cre) exacerbates acute OVA-induced eosinophilic inflammation and IL-5 production, positioning BMAL1 as a negative regulator of allergic asthma (39). Conversely, a recent study utilizing a chronic HDM model reported that myeloid BMAL1 ablation actually suppresses allergic responses, eliminating circadian differences in eosinophil infiltration and reducing airway remodeling (40). These contrasting findings are not necessarily contradictory. The OVA model typically represents an acute, highly eosinophil-driven Th2 inflammatory response, whereas the chronic HDM model better mimics the complex, mixed-granulocytic microenvironment and airway remodeling seen in humans. Myeloid BMAL1 deletion may remove immune suppression during the acute phase (exacerbating inflammation) while disrupting the metabolic adaptability of pro-fibrotic macrophages during the chronic phase (suppressing remodeling). This fundamental dichotomy carries profound clinical implications for drug development: BMAL1-targeted therapies must be stratified according to the asthma phenotype. For instance, natural bioactive compounds that enhance BMAL1 amplitude may benefit acute eosinophilic asthma but could be ineffective or even detrimental in mixed-granulocytic asthma with severe fibrosis. This context-dependency underscores that any pharmacological evaluation of BMAL1-targeting chronobiotics must be rigorously tested across multiple asthma phenotypes rather than a single model system.

Despite these well-established *in vitro* and ex vivo mechanistic cascades—linking BMAL1 deficiency to glycolytic reprogramming, HIF-1α stabilization, and NLRP3 inflammasome activation—a critical knowledge gap persists: direct *in vivo* evidence demonstrating that myeloid BMAL1 ablation in asthma models is causally responsible for subepithelial collagen deposition and fibroblast-to-myofibroblast transition (FMT) remains scarce. While the downstream mediators (e.g., IL-1β, CCL2) are established drivers of airway smooth muscle hyperplasia and fibrosis, no study has yet employed macrophage-specific BMAL1 reconstitution or inducible knockout systems to formally prove that the anti-remodeling effects of natural chronobiotics are mediated specifically through the macrophage clock. This gap is not a weakness of the existing literature but rather defines the central hypothesis that future chronopharmacological studies must rigorously test.

### 2.3 REV-ERBα dysfunction and loss of inflammatory repression

REV-ERBα functions as a primary transcriptional repressor in innate immunity. It suppresses Il6 transcription via dual mechanisms: directly binding to REV-ERB response elements (RevREs) in the Il6 promoter to recruit the NCoR-HDAC3 corepressor complex, and indirectly repressing transcription via NF-κB binding motifs (10). REV-ERBα similarly represses Ccl2 through direct promoter engagement (9). Beyond promoter binding, REV-ERBα localizes to distal enhancers selected by macrophage-lineage factors, where it suppresses gene expression by inhibiting the transcription of enhancer-derived RNAs (eRNAs) (41). Additionally, REV-ERBα directly represses p65 transcription and inhibits IκBα kinase (IKK) phosphorylation, thereby restraining IκBα degradation and preventing the nuclear translocation of the NF-κB p65 subunit (42, 43).

The repression of Ccl2 controls downstream mitogen-activated protein kinase (MAPK) signaling cascades. Decreased CCL2 secretion attenuates the activation of ERK, which regulates cell adhesion, and p38 MAPK, which regulates cell migration (9). Consequently, physiological REV-ERBα expression restricts the infiltration of circulating macrophages into inflamed lung tissues (9).

In the pulmonary microenvironment, REV-ERBα establishes the basal inflammatory threshold. Bronchial epithelial cells function as pulmonary circadian pacemakers, and the deletion of both REV-ERBα and REV-ERBβ in these cells initiates a basal inflammatory state independent of external stimulation (6). During active inflammation, stimuli dynamically regulate REV-ERBα stability. Pro-inflammatory cytokines induce the SUMOylation of REV-ERBα (6, 44), providing a structural platform for SUMO-targeted ubiquitin ligases to polyubiquitinate the receptor and target it for rapid proteasomal degradation (44, 45). Environmental factors exacerbate this pathway; for instance, REV-ERBα knockout models exposed to cigarette smoke exhibit increased neutrophil influx, elevated pro-inflammatory cytokines, and enhanced senescence markers (46). The subsequent degradation of REV-ERBα removes transcriptional repression from target genes, thereby sustaining airway inflammation and continuous immune cell recruitment (6, 46).

**Figure 1:**
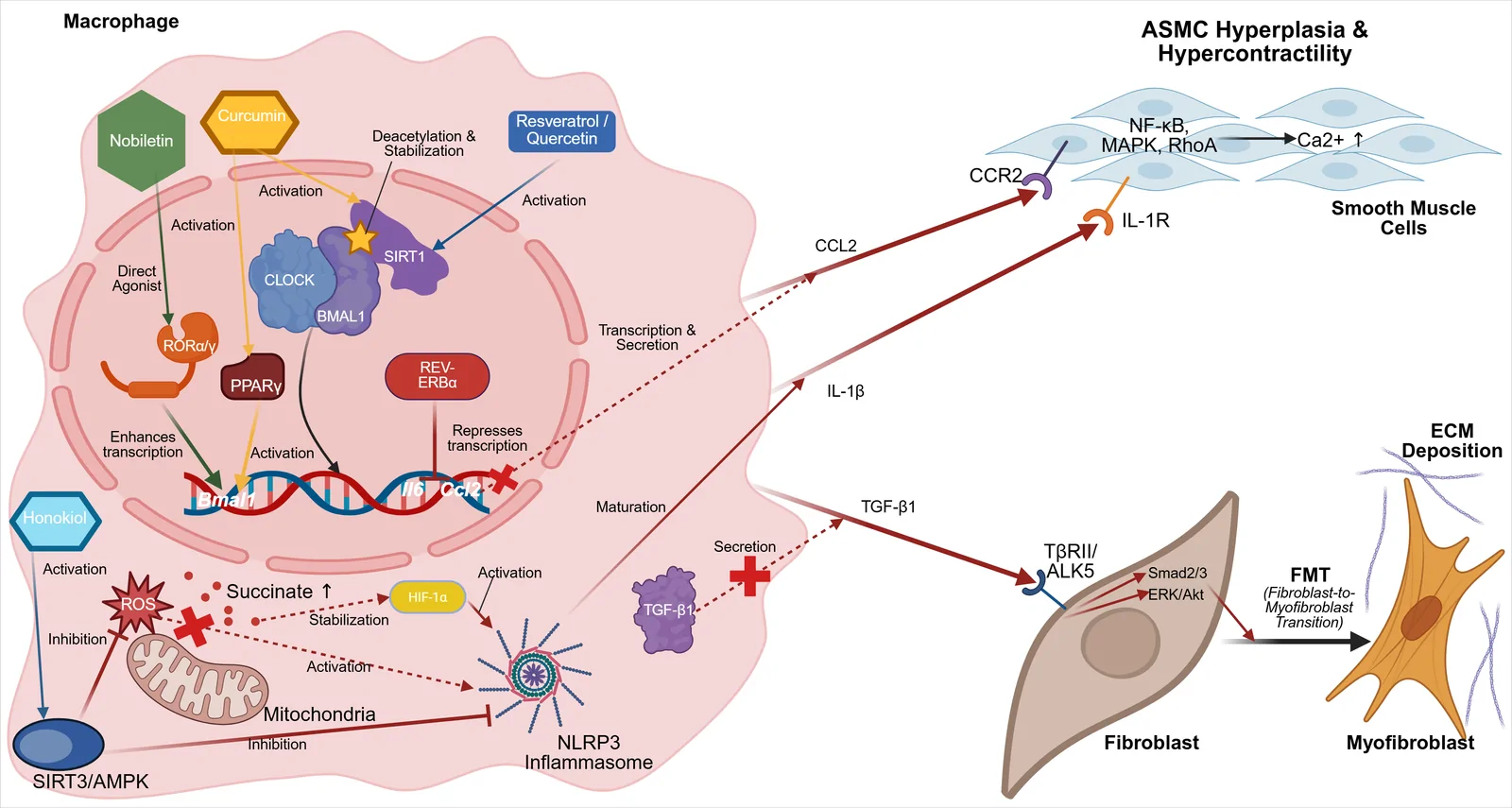
Mechanisms of targeted natural chronobiotics in modulating macrophage-stromal crosstalk and metabolic reprogramming in asthma (created with BioRender.com). Circadian disruption impairs the BMAL1/CLOCK feedback loop, leading to metabolic reprogramming characterized by succinate accumulation and HIF-1α stabilization. This metabolic shift subsequently triggers NLRP3 inflammasome activation and excessive ROS production. The resulting secretion of pro-inflammatory and pro-fibrotic factors (e.g., IL-1β, TGF-β1, CCL2) from macrophages drives ASMC hyperplasia and ECM deposition in structural cells. Natural chronobiotics exert targeted protective effects to break this pathogenic crosstalk: Curcumin activates PPARγ to enhance Bmal1 transcription; Nobiletin targets RORα/γ; Resveratrol and Quercetin activate SIRT1; and Honokiol activates the SIRT3/AMPK axis to potently inhibit ROS generation and NLRP3 activation. (Solid arrows indicate activation or promotion; red T-bars indicate inhibition).

Beyond generalized inflammatory suppression, *in vivo* asthmatic models confirm that REV-ERBα directly gates asthma-specific pathologies. Physiological airway hyperresponsiveness (AHR) exhibits strong circadian rhythmicity—peaking during the active phase—which is entirely abolished in global Rev-erbα knockout models subjected to HDM challenge, directly linking the molecular clock to airway mechanics (47). Furthermore, REV-ERBα restricts allergic pathways by directly binding to the Gata3 promoter and repressing its transcription, thereby inhibiting Th2 cell differentiation. Consequently, the administration of synthetic REV-ERB agonists (such as SR9011) has been shown to successfully mitigate OVA-induced asthmatic severity and remodeling *in vivo* (48). Together, these asthma-specific findings confirm that the disruption of the macrophage molecular clock is not merely a parallel inflammatory event, but a core driver of allergic airway hyperresponsiveness. The intricate signaling network linking macrophage circadian dysfunction to asthmatic airway remodeling, along with the targeted interventions by natural chronobiotics, is illustrated in **Figure 1**.

Collectively, these findings establish BMAL1 destabilization and REV-ERBα repression as core pathogenic nodes in the asthmatic airway. Therefore, pharmacological strategies that restore the amplitude of the macrophage clock—whether by stabilizing BMAL1, activating REV-ERBα, or modulating upstream metabolic sensors—hold therapeutic promise. The following section critically evaluates whether natural bioactive compounds can achieve such clock-directed modulation *in vivo*.

## 3 natural bioactive compounds modulating the macrophage molecular clock

The pharmacological modulation of the circadian clock represents a transition from empirical botanical medicine to targeted chronopharmacology. Crude herbal extracts comprise complex mixtures of phytochemicals, which frequently exhibit antagonistic effects on circadian rhythmicity and yield variable pharmacokinetics. Conversely, isolated natural bioactive compounds demonstrate defined structure-activity relationships. High-throughput screening combined with PER2::LUCIFERASE reporter assays indicates that specific monomers target distinct nodes within the macrophage circadian network. These monomers function by stabilizing the post-translational activation arm, enhancing transcription via direct nuclear receptor agonism, or modulating the repressive arm. We specifically selected these natural bioactive compounds—rather than generic phytochemicals—because they have demonstrated definitive clock-modulating activity in high-throughput PER2::LUCIFERASE screening assays and possess substantial *in vivo* data regarding airway remodeling and fibrosis. The detailed pharmacological profiles, including their molecular targets and current *in vivo* evidence in asthma, are systematically summarized in **Table 1**.

**Table 1:** Pharmacological mechanisms and *in vivo* asthmatic evidence of natural clock-modulating monomers.

Compound	Clock/metabolic target	*In vivo* asthma remodeling evidence	Current limitations	Macrophage clock-specific validation (e.g., cKO models)
Resveratrol	SIRT1 Activator: Deacetylates BMAL1 at K537, preventing its proteasomal degradation.	Decreases inner airway wall, mucous layer, and smooth muscle area (OVA model); alters subepithelial collagen composition; abrogates AHR.	Lacks genetic validation of clock-dependency in asthma models; exhibits ‘scaffold promiscuity’ with multiple off-target effects; low bioavailability (addressed by CD206-targeted inhalable nanocarriers; see Section 6.1).	None (Lack of specific LysM-Cre clock-knockout validation)
Quercetin	SIRT1/NRF2 Modulator & PDE4 inhibitor: Reduces intracellular ROS and preserves BMAL1 protein levels.	Directly relaxes pre-contracted airways (attenuates methacholine-induced AHR); inhibits ASMC phenotypic switching.	Direct quantitative data on subepithelial collagen deposition in allergic asthma is limited; highly pleiotropic (genetic validation and chronopharmacological dosing are required; see Section 6.2).	None
Curcumin	PPARγ Agonist (drives Bmal1 transcription) & SIRT1 Activator.	Abrogates AHR, goblet cell hyperplasia, and collagen deposition via PPARγ-dependent suppression of NF-κB and HDAC1/HIF-1α axis.	Classified as a PAINS/IMPS candidate (high chemical instability and membrane-perturbing reactivity); poor oral bioavailability (biomimetic nanocarrier co-delivery systems show promise; see Section 6.1).	None
Honokiol	SIRT3/AMPK/PGC-1α Pathway Activator: Reverses the pathological shift from OXPHOS to glycolysis.	Reduces pulmonary eosinophilic infiltration, diminishes airway remodeling, and limits macrophage accumulation via the TRPV1/NFAT pathway.	Direct interactions with core clock genes remain to be structurally elucidated; effects may be secondary to metabolic correction (spatial transcriptomics can delineate primary vs. secondary effects; see Section 6.3).	None
Nobiletin	Direct RORα/γ Agonist: Enhances Bmal1 transcription and amplifies circadian amplitude.	Demonstrates anti-fibrotic properties in bleomycin-induced pulmonary fibrosis (PI3K/AKT/mTOR). *Note: Lacks direct airway remodeling evidence in allergic asthma.*	RORα paradoxically promotes Th2 development in asthma; therapeutic effects may arise from off-target mechanisms rather than chronobiotic properties (macrophage-specific ROR knockout models are urgently needed to resolve this paradox; see Section 6.2).	None

### 3.1 Nobiletin, resveratrol, and quercetin: classical modulators of the molecular clock

The stability and transcriptional activity of the CLOCK-BMAL1 heterodimer depend on precise post-translational modifications. The CLOCK protein possesses intrinsic histone acetyltransferase activity and acetylates BMAL1 at a highly conserved lysine 537 (K537) residue (49). This acetylation facilitates CRY1 recruitment to the CLOCK-BMAL1 complex, thereby initiating the circadian repression phase. However, under oxidative stress conditions, hyperacetylation increases the vulnerability of BMAL1 to proteasomal degradation. The ubiquitin-proteasome system regulates BMAL1 protein levels through multiple E3 ubiquitin ligases, including UBE2O, UBE3A, UBR5, and STUB1 (50). During oxidative stress—such as hydrogen peroxide exposure—STUB1 undergoes nuclear translocation, colocalizes with BMAL1, and promotes the formation of Lys-48-linked polyubiquitin chains. This pathway culminates in rapid BMAL1 degradation (50).

SIRT1, an NAD^+^-dependent class III histone deacetylase, functions as a metabolic sensor that counteracts CLOCK-mediated acetylation. SIRT1 directly deacetylates BMAL1 at K537, preventing BMAL1 from entering the stress-induced ubiquitin-proteasome cascade (51). Natural polyphenols, primarily resveratrol and quercetin, modulate this axis by activating SIRT1. Mechanistically, resveratrol activates SIRT1 through multiple converging pathways (including LKB1 phosphorylation, PI3K/AKT signaling, and AMPK-mediated NAD^+^ elevation), which collectively prevent oxidative stress-induced degradation of BMAL1 and CRY1 in macrophages (52–54). However, whether this SIRT1-dependent BMAL1 stabilization represents a direct chronopharmacological action or merely an indirect consequence of generalized ROS scavenging remains contentious (see Section 5.1).

Quercetin provides comparable circadian stabilization by increasing the expression of SIRT1, BMAL1, and PER1 in lung fibroblasts (55). Additionally, it activates the Keap1/NRF2/HO-1 antioxidant axis, which lowers ROS burden and thereby limits the ROS-driven nuclear translocation of the STUB1 ubiquitin ligase that targets BMAL1 for degradation.

While resveratrol and quercetin modulate the circadian clock via post-translational protection, nobiletin directly enhances circadian transcription. High-throughput chemical screening utilizing PER2::LUCIFERASE reporter fibroblasts identified nobiletin as a potent circadian amplitude-enhancing small molecule (15, 56). Mechanistic studies demonstrate that nobiletin binds directly to the ligand-binding domains of the RORα and RORγ nuclear receptors (15), which bind to ROREs located in the Bmal1 promoter region (57).

This ROR agonism amplifies circadian amplitude and drives Nr1d1 (REV-ERBα) transcription through the feedback loop architecture, while concurrently enhancing IκBα expression to inhibit NF-κB nuclear translocation (13, 15). However, as discussed in Section 3.3, the therapeutic relevance of ROR agonism in asthma is complicated by the pro-allergic functions of RORα in Th2 cells—a paradox that demands critical scrutiny. Overall, whether through SIRT1-mediated post-translational protection (e.g., resveratrol) or direct ROR-mediated transcriptional enhancement (e.g., nobiletin), these distinct mechanisms ultimately converge on a common net effect: restoring the amplitude of the macrophage molecular clock and mitigating asthma pathophysiology.

### 3.2 Curcumin and honokiol: bridging metabolic sensors and the clock

Expanding beyond the classical flavonoids, curcumin and honokiol demonstrate robust therapeutic potential in reshaping the asthmatic microenvironment, acting through metabolic sensors intimately linked to the circadian network. Curcumin is a well-documented agonist of PPARγ, a nuclear receptor that directly binds to the PPRE elements within the Bmal1 promoter to drive its transcription (58). Additionally, curcumin activates SIRT1 to restore the amplitude of Per2 and Bmal1 oscillations in aging models (59). Crucially, *in vivo* studies using OVA-induced asthma models reveal that curcumin significantly abrogates AHR, goblet cell hyperplasia, and collagen deposition. These anti-remodeling effects are largely dependent on the PPARγ-mediated suppression of NF-κB and the HDAC1/HIF-1α signaling axis (60, 61).

Similarly, honokiol profoundly impacts macrophage immunometabolism via the AMPK/PGC-1α/SIRT3 pathway. As a direct pharmacological activator of mitochondrial SIRT3, honokiol reverses the pathological shift from oxidative phosphorylation to glycolysis, effectively inhibiting HIF-1α stabilization and NLRP3 inflammasome activation (62, 63). In allergic asthma models, honokiol administration significantly reduces pulmonary eosinophilic infiltration, diminishes airway remodeling, and limits macrophage accumulation via the TRPV1/NFAT pathway (64, 65). While direct interactions between honokiol and core clock genes remain to be structurally elucidated, its capacity to modulate the SIRT/AMPK axis functionally compensates for the metabolic dysregulation typically caused by BMAL1 deficiency.

### 3.3 The synthetic benchmark: precision agonists reveal natural limitations

To contextualize the efficacy of natural bioactive compounds, synthetic REV-ERBα agonists (e.g., SR9009, GSK4112, and STL1267) serve as critical pharmacological benchmarks. *In vivo*, precision targeting with SR9009 actively prevents bleomycin-induced collagen overexpression and attenuates abnormal epithelial-mesenchymal transition (EMT) in cigarette smoke models (12, 66). Furthermore, synthetic REV-ERB agonists robustly inhibit TGFβ1-induced FMT and strictly gate the circadian rhythmicity of AHR (67).

In stark contrast, evaluating natural product efficacy presents paradoxical challenges. For instance, while nobiletin is categorized as a RORα/γ agonist that increases Bmal1 amplitude, *in vivo* genetic evidence reveals that RORα actually promotes Th2 cell development and mucous cell hyperplasia in asthma—suggesting a ROR antagonist, rather than an agonist, would be therapeutically beneficial (68, 69). This paradox suggests that the protective effects of nobiletin and other natural polyphenols in respiratory diseases may arise from off-target mechanisms (e.g., PI3K/AKT inhibition) rather than their chronobiotic properties. This highlights a profound gap: the lack of high-affinity, structurally confirmed natural REV-ERBα agonists, and underscores the urgent need to disentangle the clock-dependent from clock-independent effects of natural therapeutics.

The nobiletin paradox—a RORα/γ agonist that enhances Bmal1 transcription yet targets a nuclear receptor that promotes Th2 differentiation—merits deeper mechanistic scrutiny. Several non-mutually exclusive explanations exist. First, RORα and RORγ exhibit cell-type-specific transcriptional programs: in Th2 cells, RORα directly activates the IL-4 and IL-13 promoters (69), whereas in macrophages, ROR agonism may primarily regulate metabolic genes (e.g., lipid metabolism and oxidative phosphorylation) rather than allergic effector functions (16). Second, the therapeutic efficacy of nobiletin in pulmonary fibrosis models was shown to be mediated through PI3K/AKT/mTOR inhibition—a pathway entirely independent of ROR transcriptional activity (70). Third, nobiletin’s broad flavonoid scaffold enables concurrent modulation of multiple kinases and transcription factors, raising the possibility that its anti-inflammatory effects in asthma are dominated by clock-independent mechanisms that override any potential pro-allergic ROR-driven signals. This paradox thus serves as a cautionary exemplar: unless genetic validation is performed, attributing its therapeutic effects to chronobiotic activity remains premature. To definitively resolve this paradox, future experimental designs must combine pharmacological intervention with genetic ablation. If nobiletin retains its anti-fibrotic efficacy in RORα-deficient (RORα-/-) macrophages, it would prove that its therapeutic effects are clock-independent (i.e., pleiotropic or off-target). Clinically, this suggests that the application of RORα agonists should be approached with caution in patients with high Th2-driven asthma, necessitating the development of dual-target strategies that can specifically uncouple their metabolic benefits from immune side effects.

## 4 *In vivo* evidence of clock-modulating monomers in asthmatic airway remodeling

The paradoxes highlighted above—particularly the disconnect between nobiletin’s ROR agonism and the pro-allergic role of RORα—underscore that *in vitro* chronobiotic activity cannot be equated with *in vivo* therapeutic efficacy. To move beyond speculative extrapolations and critically assess whether these monomers truly exert clock-dependent anti-remodeling effects, it is imperative to evaluate their *in vivo* efficacy in robust animal models of allergic asthma, with careful attention to fibrotic and hyperplastic endpoints. The transition from *in vitro* chronobiology to *in vivo* structural remodeling reveals both striking therapeutic potential and significant literature gaps.

### 4.1 Mitigating subepithelial fibrosis and collagen deposition

Subepithelial fibrosis, driven by excessive extracellular matrix (ECM) deposition and FMT, is a hallmark of asthma pathology. *In vivo* evidence strongly supports the capacity of specific natural bioactive compounds to actively reverse this structural thickening.

Resveratrol, acting through the SIRT1 axis, provides the most robust quantitative morphometric data. In OVA-induced murine asthma models, image analysis confirms that resveratrol administration significantly decreases the inner airway wall area, mucous layer area, and smooth muscle area (71). This anti-remodeling effect is structurally linked to the upregulation of PTEN and the suppression of the PI3K/Akt pathway. Furthermore, advanced Synchrotron Fourier-Transform Infrared (S-FTIR) microspectroscopy has validated that resveratrol not only decreases overall collagen volume but also fundamentally alters collagen composition within the subepithelial layers of chronically exposed asthmatic mice (72).

Similarly, curcumin and honokiol demonstrate profound *in vivo* anti-fibrotic capabilities in asthma. Chronic OVA and HDM models demonstrate that curcumin limits TGF-β secretion and substantially reduces goblet cell hyperplasia and collagen deposition via PPARγ-dependent mechanisms (60). Honokiol effectively limits macrophage accumulation in the lung interstitium and diminishes subsequent collagen deposition through the TRPV1/NFAT signaling pathway (64, 65).

A Critical Literature Gap: Despite its classification as a potent, direct RORα/γ chronobiotic, nobiletin currently lacks direct *in vivo* evidence for mitigating airway remodeling in allergic asthma. Existing *in vivo* asthmatic studies utilizing nobiletin solely report anti-inflammatory endpoints, such as the reduction of eosinophilic infiltration and eotaxin levels, without quantitative assessments of subepithelial fibrosis or FMT (73). While nobiletin has demonstrated strong anti-fibrotic properties in bleomycin-induced pulmonary fibrosis models—reducing collagen deposition via the PI3K/AKT/mTOR pathways (70)—extrapolating these interstitial fibrosis findings to the distinct microenvironment of asthmatic subepithelial remodeling remains highly speculative and requires urgent, targeted investigation.

### 4.2 Regulating airway smooth muscle hyperplasia and hyperresponsiveness

The hyper-secretion of macrophage-derived paracrine mediators (e.g., CCL2, IL-1β) directly drives airway smooth muscle cell (ASMC) proliferation and AHR. *In vivo* pharmacological data confirm that several clock-modulating monomers can intercept this pathology.

Resveratrol exhibits potent *in vivo* efficacy against AHR, with plethysmography data showing significant improvements in specific airway resistance (sRaw) and respiratory mechanics. Crucially, in OVA-challenged mice, the ability of resveratrol to abrogate AHR and mucosal hypersecretion was found to be comparable in efficacy to dexamethasone (74). Quercetin offers direct functional regulation of ASMCs; *in vivo* nebulization of quercetin attenuates methacholine-induced airway resistance, while *in vitro* mechanistic studies reveal it acts as a dual phosphodiesterase (PDE4) inhibitor and calcium channel blocker, directly relaxing pre-contracted airways (75). Recent evidence also highlights quercetin’s ability to inhibit the phenotypic switching of ASMCs from a contractile to a proliferative state by inhibiting the Wnt5a/β-catenin pathway (76).

Additionally, honokiol and curcumin have been definitively shown to reduce methacholine-responsiveness and alleviate overall AHR in OVA-challenged mice (61, 64). However, for nobiletin, the evidence gap is specific to ASMC hyperplasia: its anti-proliferative effects have only been verified in vascular and pulmonary arterial smooth muscle cells, which differ fundamentally from airway smooth muscle in developmental origin, contractile phenotype, and response to inflammatory mediators. Together with the absence of direct evidence for anti-fibrotic endpoints discussed above, this collective gap underscores that nobiletin’s purported anti-remodeling activity in asthma—whether through clock-dependent or clock-independent mechanisms—remains a hypothesis awaiting rigorous *in vivo* testing, rather than an established therapeutic effect.

## 5 The translational bottleneck: clock-dependent efficacy vs. pleiotropic effects

Although targeted natural bioactive compounds demonstrate robust *in vitro* and *in vivo* efficacy, a profound methodological challenge persists: distinguishing true clock-dependent modulation from general, clock-independent pharmacological noise.

### 5.1 The pleiotropy dilemma in natural chronobiotics

The assumption that a compound’s therapeutic efficacy stems exclusively from its ability to regulate the circadian clock is often confounded by pleiotropy and structural promiscuity. A cautionary precedent exists even in synthetic chronobiotics: SR9009, a widely used REV-ERBα agonist, has been shown to alter cellular metabolism and reduce cell viability in REV-ERBα/β double-knockout cells, indicating significant REV-ERB-independent off-target effects (77).

This specificity crisis is magnified in natural polyphenols. Resveratrol exhibits extensive “scaffold promiscuity, “ allowing it to interact non-specifically with approximately 20 distinct protein targets, with no evidence of designed polypharmacology (78, 79). Furthermore, its identity as a direct SIRT1 activator remains controversial, as initial *in vitro* validations were confounded by fluorophore artifacts, and recent clinical meta-analyses show no significant SIRT1 upregulation in human subjects (80). Similarly, curcumin is officially classified as a PAINS (pan-assay interference compounds) and IMPS (invalid metabolic panaceas) candidate. Its high chemical instability and membrane-perturbing reactivity frequently generate false-positive readouts in *in vitro* assays, making its specific circadian targets highly questionable (81, 82).

A critical methodological flaw in current literature is the bidirectional causality between the circadian clock and oxidative stress (83). Because core clock proteins are highly sensitive to ROS-induced degradation, powerful antioxidants like resveratrol and quercetin may restore BMAL1 amplitude simply by scavenging ROS and reducing the inflammatory baseline, rather than through direct pharmacological engagement of the molecular oscillator.

These confounding factors—scaffold promiscuity, assay artifacts, and the ROS-clock bidirectional causality—collectively render correlative *in vivo* observations insufficient to establish chronobiotic specificity. To definitively resolve whether a natural monomer’s therapeutic effect is genuinely clock-dependent, the field must adopt a gold-standard experimental framework that moves beyond correlation toward causation: genetic validation.

### 5.2 The genetic validation gold standard

To overcome these translational bottlenecks, the field must adopt rigorous genetic validation frameworks to prove target dependency. Among natural products, nobiletin represents a rare exception that meets this standard; its ability to enhance energy expenditure and protect against metabolic syndrome is entirely abolished in Clock mutant mice, definitively proving its clock-dependent efficacy (15).

For respiratory chronopharmacology, future research must transition from correlative observations to causal validation using macrophage-specific conditional knockout models. Specifically, we propose a multi-tiered validation framework: First, both acute (e.g., OVA) and chronic (e.g., HDM) models are required for parallel validation to exclude allergen-specific biases and address the BMAL1 dichotomy. Second, to distinguish developmental compensation effects from acute circadian functions in adult macrophages, the introduction of an inducible knockout system (e.g., LysM-CreERT2;Bmal1 fl/fl) is strongly recommended, ablating clock genes only prior to allergen challenge. Third, regarding the REV-ERBα target, given the high functional redundancy between REV-ERBα and REV-ERBβ in macrophages, robust target validation necessitates the use of Rev-erbα/β double-knockout models. A robust pharmacological validation requires demonstrating that a chronobiotic’s anti-asthmatic efficacy disappears when the target clock gene is ablated in macrophages, similar to how the anti-inflammatory effect of SR9009 on the NLRP3 inflammasome is abolished in BMAL1-deficient macrophages, and its protective effect against colitis is lost in REV-ERBα-null mice (42, 84).

However, applying this gold standard in asthma requires careful context-specific modeling. Recent evidence reveals that the role of myeloid BMAL1 is highly dichotomous: while its deletion exacerbates acute OVA-induced allergic inflammation (39), it paradoxically suppresses eosinophilic infiltration and airway remodeling in chronic HDM-induced models (40). Therefore, true chrono-systems pharmacology must not only validate the physical target engagement of natural bioactive compounds (e.g., via CETSA) but also rigorously test their clock-dependent efficacy across diverse, clinically relevant asthmatic phenotypes utilizing targeted genetic ablation.

## 6 Future perspectives and concluding remarks

**Figure 2:**
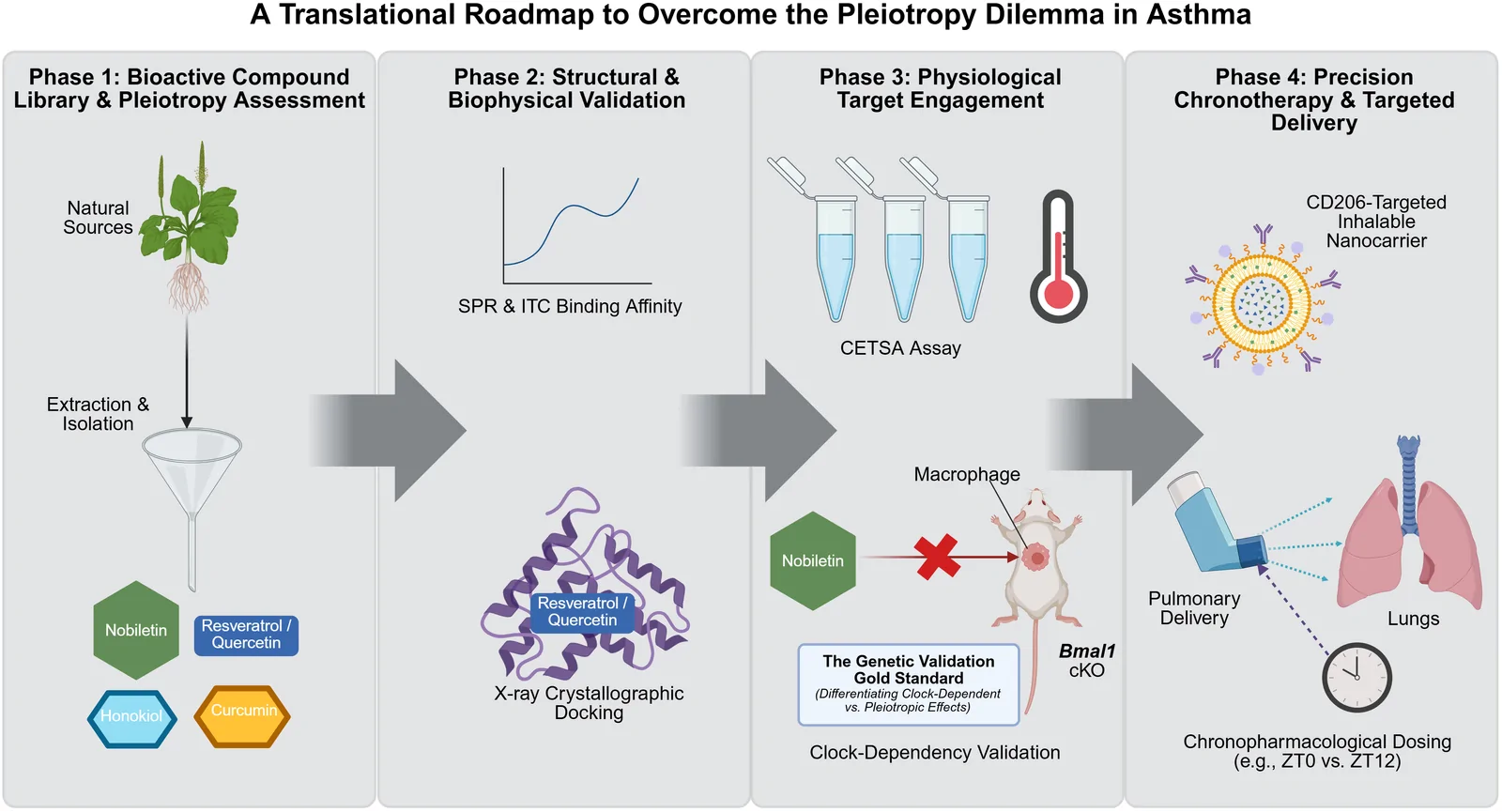
A translational roadmap to overcome the pleiotropy dilemma of natural chronobiotics in asthma (created with BioRender.com). This pipeline illustrates a rigorous, four-phase approach to transition natural bioactive compounds from bench to bedside. Phase 1: Establishment of a comprehensive bioactive compound library for unbiased evaluation of pleiotropic versus clock-dependent effects. Phase 2: Structural and biophysical validation of compound-target interactions (e.g., molecular docking, SPR). Phase 3: Application of the genetic validation “gold standard” using macrophage-specific Bmal1 conditional knockout murine models to effectively distinguish clock-dependent therapeutic efficacies from pleiotropic noise. Phase 4: Implementation of precision chronotherapy integrating CD206-targeted inhalable nanocarriers and chronopharmacological dosing strategies (e.g., optimizing administration at ZT0 vs. ZT12) to maximize target engagement and minimize off-target effects.

To transition from the current limitations of natural chronobiotics to clinically viable respiratory therapies, the field must adopt a multidisciplinary translational framework. Future research must overcome pharmacokinetic barriers, optimize temporal dosing, and precisely map the spatial dynamics of the asthmatic niche. A comprehensive translational roadmap designed to overcome the pleiotropy dilemma and these translational bottlenecks is presented in **Figure 2**.

### 6.1 Overcoming pharmacokinetic barriers: CD206-targeted inhalable nanocarriers

Despite their robust *in vitro* chronobiotic mechanisms, natural polyphenols like resveratrol and quercetin suffer from extensive intestinal and hepatic first-pass metabolism, yielding an oral bioavailability of typically less than 1%. To achieve therapeutic concentrations within the pulmonary microenvironment, localized inhalation utilizing advanced nanocarriers is essential.

Recent advancements in nanomedicine provide a blueprint for overcoming this barrier. Formulations such as lipid-core nanocapsules and spray-dried proliposomes have been shown to significantly enhance the deep-lung deposition, localized retention, and anti-inflammatory efficacy of resveratrol and curcumin *in vivo* (85, 86). However, the most critical evolution for asthma chronotherapy is the development of macrophage-targeted inhalable platforms. Pulmonary macrophages highly express the mannose receptor (MR/CD206). Recent studies demonstrate that decorating lipid-polymer hybrid nanoparticles with D-mannose or trimannose conjugates allows for the highly selective, receptor-mediated endocytosis of therapeutics directly into CD206+ alveolar and interstitial macrophages (87, 88).

Furthermore, biomimetic nanotechnology offers unprecedented advantages for natural chronobiotics. Platelet membrane-coated PLGA nanoparticles, as well as M2 macrophage membrane-derived decoy nanovesicles, have been recently utilized to co-load polyphenols (e.g., curcumin and resveratrol) for inhalation (89, 90). These biomimetic coatings facilitate targeted homing to inflamed lung tissues, promote immune evasion, and dramatically enhance the local macrophage reprogramming capacity. Integrating targeted chronobiotics with CD206-directed or biomimetic inhalable nanocarriers represents the most promising avenue to resolve the bioavailability bottleneck.

Nevertheless, the clinical translation of inhalable nanocarriers must address several practical obstacles. First, the differential expression of CD206 on M1 and M2 macrophages complicates targeting; while targeting CD206 may successfully deliver therapeutics to pro-fibrotic M2-like macrophages, it might inadvertently bypass pro-inflammatory M1-like macrophages, thereby requiring highly refined dual-ligand designs. Second, pulmonary toxicity from long-term inhalable nanoparticle administration and the physical barrier posed by airway mucociliary clearance—which is often hyperactive in asthma—remain significant hurdles. Third, because the current efficacy data for these natural bioactive compounds are predominantly based on oral administration, transitioning to inhalation requires establishing precise new dose-response relationships to prevent non-specific cytotoxicity caused by sudden localized concentration spikes in the lung. Rigorous long-term biocompatibility and biodistribution studies in clinically relevant chronic asthma models are prerequisite before these platforms can be integrated into chronotherapeutic regimens.

### 6.2 Chronopharmacological dosing: precision timing of intervention

Asthma pathophysiology is fundamentally gated by the circadian clock. For example, physiological AHR exhibits profound day-night variations—peaking at the onset of the active phase in murine models—a phenomenon entirely abolished in Rev-erbα deficient mice (47). Therefore, the efficacy of chronobiotics is not static but highly dependent on the temporal phase of administration.

Chronopharmacology dictates that aligning drug administration with specific circadian phases (Zeitgeber Times, ZT) maximizes therapeutic indices. Even standard synthetic anti-inflammatories exhibit strict temporal dependencies; inhaled dexamethasone administered at ZT0 (the onset of the resting phase in mice) more effectively suppresses mucus-related gene expression (e.g., Clca3) and alters the phase of the pulmonary clock compared to administration at ZT18 (91). For natural clock modulators, emerging *in vivo* studies utilizing synthetic ROR ligands (e.g., SR1001) in asthmatic models underscore that restoring macrophage clock function resolves inflammation most efficiently when synced with endogenous circadian peaks (26, 92). Future *in vivo* studies must mandate administration time (e.g., ZT0 vs. ZT12) as an independent experimental variable, rather than a *post-hoc* consideration. Systematic time-course pharmacokinetic/pharmacodynamic (PK/PD) modeling, coupled with circadian-phase-specific outcome measurements (e.g., AHR at CT8 vs. CT20), is required to establish evidence-based chronotherapy protocols that maximize the therapeutic window of natural chronobiotics while minimizing off-target chronotoxicity.

### 6.3 Mapping the asthmatic niche: spatial transcriptomics and crosstalk

While traditional bulk RNA sequencing has illuminated global pulmonary inflammation, it obscures the localized physical interactions driving airway remodeling. The latest single-cell and spatial transcriptomic maps of the human airway wall reveal that asthma is not a homogenous inflammatory state, but is rather driven by discrete “cellular ecosystems” or spatial niches characterized by intensive immune-stromal proximity (93).

Within these specific niches—such as the adventitial cuff and subepithelial layers—macrophages undergo significant transcriptional reprogramming. Recent single-cell RNA sequencing (scRNA-seq) in allergic asthma models directly implicates specific macrophage-fibroblast circuits. Monocyte-derived CCR2+ macrophages interact tightly with myofibroblast subpopulations via specific paracrine signaling axes, notably IL1B, CXCL12/CXCR4, and CCL8/CCR2, to jointly drive pathological ECM deposition (94, 95). Future chronobiotic research must overlay these spatial transcriptomic coordinates with circadian readouts. By pinpointing exactly which spatial niches house clock-dysregulated macrophages, researchers can utilize spatial multi-omics to dynamically trace how natural bioactive compounds disrupt these localized macrophage-fibroblast interactions and dismantle the pro-fibrotic niche *in situ*.

Critically, spatial transcriptomics offers a unique opportunity to resolve the central dilemma of this review—distinguishing clock-dependent from clock-independent mechanisms. By spatially co-registering core clock genes (e.g., Bmal1, Rev-erbα) with fibrotic markers (e.g., Col1a1, Acta2) and inflammatory mediators (e.g., Il1b, Ccl2) within discrete anatomical compartments, researchers can determine whether natural chronobiotics exert their anti-remodeling effects preferentially in clock-intact macrophage niches—supporting a clock-dependent mechanism—or equally in clock-deficient niches, which would indicate a clock-independent, generalized anti-inflammatory action. Such spatial resolution would transform the current correlative observations into causal, spatially anchored mechanistic insights, directly informing whether future drug development should prioritize ROR agonism, SIRT1 activation, or broad-spectrum antioxidant optimization.

### 6.4 Concluding remarks

The intrinsic molecular clock within pulmonary macrophages acts as a critical gatekeeper of the asthmatic microenvironment. The degradation of key circadian repressors (REV-ERBα) and the suppression of metabolic sensors (BMAL1) initiate a cascade of glycolytic reprogramming and paracrine hypersecretion (e.g., TGF-β1, IL-1β, CCL2). This dysregulation inextricably links circadian disruption to the structural hallmarks of asthma: subepithelial collagen deposition and airway smooth muscle hyperplasia.

Targeting this network with natural chronobiotics—such as the SIRT1-activator resveratrol or the ROR-agonist nobiletin—offers a compelling strategy to restore immune-stromal homeostasis. However, advancing this field requires abandoning speculative *in vitro* extrapolations. The true chrono-therapeutic potential of these monomers must be rigorously disentangled from their pleiotropic, non-specific antioxidant effects. By demanding genetic validation through macrophage-specific clock-knockout models, optimizing localized delivery via CD206-targeted nanocarriers, and mapping efficacy through spatial transcriptomics, future research can definitively translate natural chronobiotics into disease-modifying therapies for nocturnal asthma and airway remodeling. Without such rigorous frameworks, the field risks perpetuating correlative inferences that confound clock-dependent pharmacology with generalized antioxidant effects.

## References

[1] Scheer FAJL Hilton MF Evoniuk HL Shiels SA Malhotra A Sugarbaker R . The endogenous circadian system worsens asthma at night independent of sleep and other daily behavioral or environmental cycles. Proc Natl Acad Sci. (2021) 118:e2018486118. doi: 10.1073/pnas.2018486118 34493686 PMC8449316

[2] Durrington HJ Farrow SN Loudon AS Ray DW . The circadian clock and asthma. Thorax. (2013) 69:90–2. doi: 10.1136/thoraxjnl-2013-203482 23704227

[3] Burioka N Fukuoka Y Koyanagi S Miyata M Takata M Chikumi H . Asthma: Chronopharmacotherapy and the molecular clock. Adv Drug Delivery Rev. (2010) 62:946–55. doi: 10.1016/j.addr.2010.03.012 20359514

[4] Barnes PJ . Inhaled glucocorticoids for asthma. N Engl J Med. (1995) 332:868–75. doi: 10.1056/NEJM199503303321307 7870143

[5] Holimon TD Chafin CC Self TH . Nocturnal asthma uncontrolled by inhaled corticosteroids. Drugs. (2001) 61:391–418. doi: 10.2165/00003495-200161030-00007 11293649

[6] Pariollaud M Gibbs JE Hopwood TW Brown S Begley N Vonslow R . Circadian clock component REV-ERBα controls homeostatic regulation of pulmonary inflammation. J Clin Invest. (2018) 128:2281–96. doi: 10.1172/JCI93910 29533925 PMC5983347

[7] Timmons GA Carroll RG O Siorain JR Cervantes-Silva MP Fagan LE Cox SL . The circadian clock protein BMAL1 acts as a metabolic sensor in macrophages to control the production of pro IL-1β. Front Immunol. (2021) 12:700431. doi: 10.3389/fimmu.2021.700431 34858390 PMC8630747

[8] O'Siorain JR Cox SL Payet C Nally FK He Y Drewinksi TT . Time‐of‐day control of mitochondria regulates NLRP3 inflammasome activation in macrophages. FASEB J. (2024) 38:e70235. doi: 10.1096/fj.202400508RR 39686706 PMC11669068

[9] Sato S Sakurai T Ogasawara J Takahashi M Izawa T Imaizumi K . A circadian clock gene, Rev-erbα, modulates the inflammatory function of macrophages through the negative regulation of Ccl2 expression. J Immunol. (2014) 192:407–17. doi: 10.4049/jimmunol.1301982 24307731

[10] Sato S Sakurai T Ogasawara J Shirato K Ishibashi Y Oh-ishi S . Direct and indirect suppression of interleukin-6 gene expression in murine macrophages by nuclear orphan receptor REV-ERBα. Sci World J. (2014) 2014:1–10. doi: 10.1155/2014/685854 25401152 PMC4220616

[11] Cunningham PS Meijer P Nazgiewicz A Anderson SG Borthwick LA Bagnall J . The circadian clock protein REVERBα inhibits pulmonary fibrosis development. Proc Natl Acad Sci. (2020) 117:1139–47. doi: 10.1073/pnas.1912109117 31879343 PMC6969503

[12] Wang Q Sundar IK Lucas JH Muthumalage T Rahman I . Molecular clock REV-ERBα regulates cigarette smoke–induced pulmonary inflammation and epithelial-mesenchymal transition. JCI Insight. (2021) 6:e145200. doi: 10.1172/jci.insight.145200 34014841 PMC8262497

[13] Shinozaki A Misawa K Ikeda Y Haraguchi A Kamagata M Tahara Y . Potent effects of flavonoid nobiletin on amplitude, period, and phase of the circadian clock rhythm in PER2::LUCIFERASE mouse embryonic fibroblasts. PloS One. (2017) 12:e170904. doi: 10.1371/journal.pone.0170904 28152057 PMC5289493

[14] Xu T Lu B . The effects of phytochemicals on circadian rhythm and related diseases. Crit Rev Food Sci Nutr. (2019) 59:882–92. doi: 10.1080/10408398.2018.1493678 29999408

[15] He B Nohara K Park N Park Y Guillory B Zhao Z . The small molecule nobiletin targets the molecular oscillator to enhance circadian rhythms and protect against metabolic syndrome. Cell Metab. (2016) 23:610–21. doi: 10.1016/j.cmet.2016.03.007 27076076 PMC4832569

[16] Spaleniak W Cuendet M . Resveratrol as a circadian clock modulator: mechanisms of action and therapeutic applications. Mol Biol Rep. (2023) 50:6159–70. doi: 10.1007/s11033-023-08513-2 37231216 PMC10289927

[17] Allada R Bass J . Circadian mechanisms in medicine. N Engl J Med. (2021) 384:550–61. doi: 10.1056/NEJMra1802337 33567194 PMC8108270

[18] Liu Y Sancar A . Biochemical mechanism of the mammalian circadian clock. FEBS Lett. (2026) 600:716–31. doi: 10.1002/1873-3468.70150 40854106 PMC12380416

[19] Kavakli IH Ozturk N Baris I . Protein interaction networks of the mammalian core clock proteins. Adv Protein Chem Struct Biol. (2022) 131:207–33. doi: 10.1016/bs.apcsb.2022.04.001 35871891

[20] Kim E Yoo S Chen Z . Circadian stabilization loop: the regulatory hub and therapeutic target promoting circadian resilience and physiological health. F1000Research. (2022) 11:1236. doi: 10.12688/f1000research.126364.2 36415204 PMC9652504

[21] Zhang Y Fang B Emmett MJ Damle M Sun Z Feng D . Discrete functions of nuclear receptor Rev-erbα couple metabolism to the clock. Science. (2015) 348:1488–92. doi: 10.1126/science.aab3021 26044300 PMC4613749

[22] Gibbs JE Blaikley J Beesley S Matthews L Simpson KD Boyce SH . The nuclear receptor REV-ERBα mediates circadian regulation of innate immunity through selective regulation of inflammatory cytokines. Proc Natl Acad Sci. (2012) 109:582–7. doi: 10.1073/pnas.1106750109 22184247 PMC3258648

[23] Collins EJ Cervantes-Silva MP Timmons GA O'Siorain JR Curtis AM Hurley JM . Post-transcriptional circadian regulation in macrophages organizes temporally distinct immunometabolic states. Genome Res. (2021) 31:171–85. doi: 10.1101/gr.263814.120 33436377 PMC7849412

[24] Sajti E Link VM Ouyang Z Spann NJ Westin E Romanoski CE . Transcriptomic and epigenetic mechanisms underlying myeloid diversity in the lung. Nat Immunol. (2020) 21:221–31. doi: 10.1038/s41590-019-0582-z 31959980 PMC7667722

[25] Aegerter H Lambrecht BN Jakubzick CV . Biology of lung macrophages in health and disease. Immunity. (2022) 55:1564–80. doi: 10.1016/j.immuni.2022.08.010 36103853 PMC9533769

[26] Teppan J Schwanzer J Rittchen S Bärnthaler T Lindemann J Nayak B . The disrupted molecular circadian clock of monocytes and macrophages in allergic inflammation. Front Immunol. (2024) 15:1408772. doi: 10.3389/fimmu.2024.1408772 38863703 PMC11165079

[27] Mills EL O'Neill LA . Reprogramming mitochondrial metabolism in macrophages as an anti‐inflammatory signal. Eur J Immunol. (2016) 46:13–21. doi: 10.1002/eji.201445427 26643360

[28] Tannahill GM Curtis AM Adamik J Palsson-McDermott EM McGettrick AF Goel G . Succinate is an inflammatory signal that induces IL-1β through HIF-1α. Nature. (2013) 496:238–42. doi: 10.1038/nature11986 23535595 PMC4031686

[29] Corcoran SE O'Neill LAJ . HIF1α and metabolic reprogramming in inflammation. J Clin Invest. (2016) 126:3699–707. doi: 10.1172/JCI84431 27571407 PMC5096812

[30] Qiu B Yuan P Du X Jin H Du J Huang Y . Hypoxia inducible factor-1α is an important regulator of macrophage biology. Heliyon. (2023) 9:e17167. doi: 10.1016/j.heliyon.2023.e17167 37484306 PMC10361316

[31] Alexander RK Liou Y Knudsen NH Starost KA Xu C Hyde AL . Bmal1 integrates mitochondrial metabolism and macrophage activation. Elife. (2020) 9:e54090. doi: 10.7554/eLife.54090 32396064 PMC7259948

[32] Mills EL Kelly B Logan A Costa ASH Varma M Bryant CE . Succinate dehydrogenase supports metabolic repurposing of mitochondria to drive inflammatory macrophages. Cell. (2016) 167:457–70. doi: 10.1016/j.cell.2016.08.064 27667687 PMC5863951

[33] Casey AM Murphy MP . Uncovering the source of mitochondrial superoxide in pro-inflammatory macrophages: Insights from immunometabolism. Biochim Et Biophys Acta (Bba) - Mol Basis Dis. (2022) 1868:166481. doi: 10.1016/j.bbadis.2022.166481 35792320 PMC7614207

[34] Early JO Menon D Wyse CA Cervantes-Silva MP Zaslona Z Carroll RG . Circadian clock protein BMAL1 regulates IL-1β in macrophages via NRF2. Proc Natl Acad Sci. (2018) 115:E8460–8. doi: 10.1073/pnas.1800431115 30127006 PMC6130388

[35] Kobayashi EH Suzuki T Funayama R Nagashima T Hayashi M Sekine H . Nrf2 suppresses macrophage inflammatory response by blocking proinflammatory cytokine transcription. Nat Commun. (2016) 7:11624. doi: 10.1038/ncomms11624 27211851 PMC4879264

[36] Atallah R Gindlhuber J Heinemann A . Succinate in innate immunity: Linking metabolic reprogramming to immune modulation. Front Immunol. (2025) 16:1661948. doi: 10.3389/fimmu.2025.1661948 41080558 PMC12507857

[37] Shim D Eo J Kim S Hwang I Nam B Shin J . Deficiency of circadian clock gene Bmal1 exacerbates noncanonical inflammasome-mediated pyroptosis and lethality via Rev-erbα-C/EBPβ-SAA1 axis. Exp Mol Med. (2024) 56:370–82. doi: 10.1038/s12276-024-01162-w 38297162 PMC10907614

[38] Xiang X Zhong C Tang H . The role of BMAL1 in regulating circadian rhythms during airway remodeling in asthma. J Immunol Res. (2026) 2026:e6983721. doi: 10.1155/jimr/6983721 42274140 PMC13255153

[39] Zasłona Z Case S Early JO Lalor SJ McLoughlin RM Curtis AM . The circadian protein BMAL1 in myeloid cells is a negative regulator of allergic asthma. Am J Physiol Lung Cell Mol Physiol. (2017) 312:L855–60. doi: 10.1152/ajplung.00072.2017 28336811

[40] Hong H Zhang J Cao X Wu Y Chan TF Tian XY . Myeloid Bmal1 deletion suppresses the house dust mite-induced chronic lung allergy. J Leukoc Biol. (2024) 115:164–76. doi: 10.1093/jleuko/qiad047 37170891

[41] Lam MTY Cho H Lesch HP Gosselin D Heinz S Tanaka-Oishi Y . Rev-Erbs repress macrophage gene expression by inhibiting enhancer-directed transcription. Nature. (2013) 498:511–5. doi: 10.1038/nature12209 23728303 PMC3839578

[42] Wang S Lin Y Yuan X Li F Guo L Wu B . REV-ERBα integrates colon clock with experimental colitis through regulation of NF-κB/NLRP3 axis. Nat Commun. (2018) 9:4246. doi: 10.1038/s41467-018-06568-5 30315268 PMC6185905

[43] Guo D Zhu Y Sun H Xu X Zhang S Hao Z . Pharmacological activation of REV-ERBα represses LPS-induced microglial activation through the NF-κB pathway. Acta Pharmacol Sin. (2019) 40:26–34. doi: 10.1038/s41401-018-0064-0 29950615 PMC6318300

[44] Chang H Yeh ETH . SUMO: From bench to bedside. Physiol Rev. (2020) 100:1599–619. doi: 10.1152/physrev.00025.2019 32666886 PMC7717128

[45] Miteva M Keusekotten K Hofmann K Praefcke GJK Dohmen RJ . Sumoylation as a signal for polyubiquitylation and proteasomal degradation. Sub-cell Biochem. (2010) 54:195–214. doi: 10.1007/978-1-4419-6676-6_16 21222284

[46] Sundar IK Rashid K Sellix MT Rahman I . The nuclear receptor and clock gene REV-ERBα regulates cigarette smoke-induced lung inflammation. Biochem Biophys Res Commun. (2017) 493:1390–5. doi: 10.1016/j.bbrc.2017.09.157 28974420 PMC5756581

[47] Durrington HJ Krakowiak K Meijer P Begley N Maidstone R Goosey L . Circadian asthma airway responses are gated by REV-ERBα. Eur Respir J. (2020) 56:1902407. doi: 10.1183/13993003.02407-2019 32586876 PMC7613655

[48] Tiwari D Ahuja N Kumar S Kalra R Nanduri R Gupta S . Nuclear receptor Nr1d1 alleviates asthma by abating GATA3 gene expression and Th2 cell differentiation. Cell Mol Life Sci Cmls. (2022) 79:308. doi: 10.1007/s00018-022-04323-0 35596832 PMC11073070

[49] Hirayama J Sahar S Grimaldi B Tamaru T Takamatsu K Nakahata Y . CLOCK-mediated acetylation of BMAL1 controls circadian function. Nature. (2007) 450:1086–90. doi: 10.1038/nature06394 18075593

[50] Ullah K Chen S Lu J Wang X Liu Q Zhang Y . The E3 ubiquitin ligase STUB1 attenuates cell senescence by promoting the ubiquitination and degradation of the core circadian regulator BMAL1. J Biol Chem. (2020) 295:4696–708. doi: 10.1074/jbc.RA119.011280 32041778 PMC7135990

[51] Nakahata Y Kaluzova M Grimaldi B Sahar S Hirayama J Chen D . The NAD+-dependent deacetylase SIRT1 modulates CLOCK-mediated chromatin remodeling and circadian control. Cell. (2008) 134:329–40. doi: 10.1016/j.cell.2008.07.002 18662547 PMC3526943

[52] Huang Y Lu J Zhan L Wang M Shi R Yuan X . Resveratrol-induced Sirt1 phosphorylation by LKB1 mediates mitochondrial metabolism. J Biol Chem. (2021) 297:100929. doi: 10.1016/j.jbc.2021.100929 34216621 PMC8326426

[53] Zong Y Sun L Liu B Deng Y Zhan D Chen Y . Resveratrol inhibits LPS-induced MAPKs activation via activation of the phosphatidylinositol 3-kinase pathway in murine RAW 264.7 macrophage cells. PloS One. (2012) 7:e44107. doi: 10.1371/journal.pone.0044107 22952890 PMC3432093

[54] Tan X Li L Wang J Zhao B Pan J Wang L . Resveratrol prevents acrylamide-induced mitochondrial dysfunction and inflammatory responses via targeting circadian regulator Bmal1 and Cry1 in hepatocytes. J Agric Food Chem. (2019) 67:8510–9. doi: 10.1021/acs.jafc.9b03368 31294559

[55] Okada Y Okada M . Quercetin, caffeic acid and resveratrol regulate circadian clock genes and aging-related genes in young and old human lung fibroblast cells. Mol Biol Rep. (2020) 47:1021–32. doi: 10.1007/s11033-019-05194-8 31773385

[56] Chen Z Yoo S Park Y Kim K Wei S Buhr E . Identification of diverse modulators of central and peripheral circadian clocks by high-throughput chemical screening. Proc Natl Acad Sci. (2012) 109:101–6. doi: 10.1073/pnas.1118034108 22184224 PMC3252927

[57] Takeda Y Jothi R Birault V Jetten AM . RORγ directly regulates the circadian expression of clock genes and downstream targets *in vivo*. Nucleic Acids Res. (2012) 40:8519–35. doi: 10.1093/nar/gks630 22753030 PMC3458568

[58] Chen F Guo N Cao G Zhou J Yuan Z . Molecular analysis of curcumin-induced polarization of murine RAW264.7 macrophages. J Cardiovasc Pharmacol. (2014) 63:544–52. doi: 10.1097/FJC.0000000000000079 24709638

[59] Kukkemane K Jagota A . Therapeutic effects of curcumin on age-induced alterations in daily rhythms of clock genes and Sirt1 expression in the SCN of male Wistar rats. Biogerontology. (2019) 20:405–19. doi: 10.1007/s10522-018-09794-y 30607623

[60] Zhu T Chen Z Chen G Wang D Tang S Deng H . Curcumin attenuates asthmatic airway inflammation and mucus hypersecretion involving a PPARγ-dependent NF-κB signaling pathway *in vivo* and *in vitro*. Mediators Inflammation. (2019) 2019:4927430. doi: 10.1155/2019/4927430 31073274 PMC6470457

[61] Islam R Dash D Singh R . An antioxidant ameliorates allergic airway inflammation by inhibiting HDAC 1 via HIF-1α/VEGF axis suppression in mice. Sci Rep. (2023) 13:9637. doi: 10.1038/s41598-023-36678-0 37316684 PMC10267105

[62] Pillai VB Samant S Sundaresan NR Raghuraman H Kim G Bonner MY . Honokiol blocks and reverses cardiac hypertrophy in mice by activating mitochondrial Sirt3. Nat Commun. (2015) 6:6656. doi: 10.1038/ncomms7656 25871545 PMC4441304

[63] Cai X Jiang X Zhao M Su K Tang M Hong F . Identification of the target protein and molecular mechanism of honokiol in anti-inflammatory action. Phytomedicine Int J Phytotherapy Phytopharmacology. (2023) 109:154617. doi: 10.1016/j.phymed.2022.154617 36610140

[64] Munroe ME Businga TR Kline JN Bishop GA . Anti-inflammatory effects of the neurotransmitter agonist honokiol in a mouse model of allergic asthma. J Immunol (Baltimore Md 1950). (2010) 185:5586–97. doi: 10.4049/jimmunol.1000630 20889543 PMC3197781

[65] Tu L Zhu X Peng M Li H Wang Y Liu J . Magnolia officinalis Rehder & E. Wilson extract and its main component honokiol alleviate asthma by reducing respiratory inflammation through the TRPV1/NFAT/TSLP pathway. Phytomedicine Int J Phytotherapy Phytopharmacology. (2026) 152:157840. doi: 10.1016/j.phymed.2026.157840 41570784

[66] Wang Q Sundar IK Lucas JH Park J Nogales A Martinez-Sobrido L . Circadian clock molecule REV-ERBα regulates lung fibrotic progression through collagen stabilization. Nat Commun. (2023) 14:1295. doi: 10.1038/s41467-023-36896-0 36894533 PMC9996598

[67] Prasad C Hahn K Duraisamy SK Salathe MA Huang SK Burris TP . Rev-erbα agonists suppresses TGFβ1-induced fibroblast-to-myofibroblast transition and pro-fibrotic phenotype in human lung fibroblasts. Biochem Biophys Res Commun. (2023) 669:120–7. doi: 10.1016/j.bbrc.2023.05.092 37269594 PMC11034855

[68] Jaradat M Stapleton C Tilley SL Dixon D Erikson CJ McCaskill JG . Modulatory role for retinoid-related orphan receptor alpha in allergen-induced lung inflammation. Am J Respir Crit Care Med. (2006) 174:1299–309. doi: 10.1164/rccm.200510-1672OC 16973978 PMC2648295

[69] Roberts J Chevalier A Hawerkamp HC Yeow A Matarazzo L Schwartz C . Retinoic acid-related orphan receptor α is required for generation of Th2 cells in type 2 pulmonary inflammation. J Immunol (Baltimore Md 1950). (2023) 211:626–32. doi: 10.4049/jimmunol.2200896 37387671 PMC10404816

[70] Yu S Liu K Zhou F Yang X Zhang J Yu K . Nobiletin from Citrus reticulata Blanco alleviates pulmonary fibrosis through inhibiting the PI3K/AKT pathway and epithelial-mesenchymal transition. J Ethnopharmacol. (2025) 349:119965. doi: 10.1016/j.jep.2025.119965 40354836

[71] Chen G Tang J Ni Z Chen Q Li Z Yang W . Antiasthmatic effects of resveratrol in ovalbumin-induced asthma model mice involved in the upregulation of PTEN. Biol Pharm Bull. (2015) 38:507–13. doi: 10.1248/bpb.b14-00610 25739523

[72] Mazarakis N Vongsvivut J Bambery KR Ververis K Tobin MJ Royce SG . Investigation of molecular mechanisms of experimental compounds in murine models of chronic allergic airways disease using synchrotron Fourier-transform infrared microspectroscopy. Sci Rep. (2020) 10:11713. doi: 10.1038/s41598-020-68671-2 32678217 PMC7366655

[73] Wu Y Zhou C Tao J Li S . Antagonistic effects of nobiletin, a polymethoxyflavonoid, on eosinophilic airway inflammation of asthmatic rats and relevant mechanisms. Life Sci. (2006) 78:2689–96. doi: 10.1016/j.lfs.2005.10.029 16337971

[74] Lee M Kim S Kwon O Oh S Lee H Ahn K . Anti-inflammatory and anti-asthmatic effects of resveratrol, a polyphenolic stilbene, in a mouse model of allergic asthma. Int Immunopharmacol. (2009) 9:418–24. doi: 10.1016/j.intimp.2009.01.005 19185061

[75] Townsend EA Emala CWS . Quercetin acutely relaxes airway smooth muscle and potentiates β-agonist-induced relaxation via dual phosphodiesterase inhibition of PLCβ and PDE4. Am J Physiol Lung Cell Mol Physiol. (2013) 305:L396–403. doi: 10.1152/ajplung.00125.2013 23873842 PMC3763034

[76] Zhou H Lai Y Zhu Y Shao F Ma G Yang N . Quercetin improves airway remodeling in COPD rats by suppressing phenotypic switch of ASMCs via inhibiting the Wnt5a/β-catenin pathway. Phytomedicine Int J Phytotherapy Phytopharmacology. (2025) 139:156491. doi: 10.1016/j.phymed.2025.156491 39955824

[77] Dierickx P Emmett MJ Jiang C Uehara K Liu M Adlanmerini M . SR9009 has REV-ERB-independent effects on cell proliferation and metabolism. Proc Natl Acad Sci USA. (2019) 116:12147–52. doi: 10.1073/pnas.1904226116 31127047 PMC6589768

[78] Saqib U Kelley TT Panguluri SK Liu D Savai R Baig MS . Polypharmacology or promiscuity? Structural interactions of resveratrol with its bandwagon of targets. Front Pharmacol. (2018) 9:1201. doi: 10.3389/fphar.2018.01201 30405416 PMC6207623

[79] Britton RG Kovoor C Brown K . Direct molecular targets of resveratrol: identifying key interactions to unlock complex mechanisms. Ann N Y Acad Sci. (2015) 1348:124–33. doi: 10.1111/nyas.12796 26099829

[80] Mansouri F Feliziani G Bordoni L Gabbianelli R . Impact of resveratrol supplementation on human sirtuin 1: a grading of recommendations assessment, development and evaluation-assessed systematic review and dose-response meta-analysis of randomized controlled trials. J Acad Nutr Diet. (2025) 125:1299–314. doi: 10.1016/j.jand.2025.03.011 40158656

[81] Nelson KM Dahlin JL Bisson J Graham J Pauli GF Walters MA . The essential medicinal chemistry of curcumin. J Med Chem. (2017) 60:1620–37. doi: 10.1021/acs.jmedchem.6b00975 28074653 PMC5346970

[82] Bolger GT Pucaj K Minta YO Sordillo P . Relationship between the *in vitro* efficacy, pharmacokinetics and *in vivo* efficacy of curcumin. Biochem Pharmacol. (2022) 205:115251. doi: 10.1016/j.bcp.2022.115251 36130650

[83] Liu F Zhang X Zhao B Tan X Wang L Liu X . Role of food phytochemicals in the modulation of circadian clocks. J Agric Food Chem. (2019) 67:8735–9. doi: 10.1021/acs.jafc.9b02263 31244204

[84] Hong H Cheung YM Cao X Wu Y Li C Tian XY . REV-ERBα agonist SR9009 suppresses IL-1β production in macrophages through BMAL1-dependent inhibition of inflammasome. Biochem Pharmacol. (2021) 192:114701. doi: 10.1016/j.bcp.2021.114701 34324866

[85] de Oliveira MTP Coutinho DDS Guterres SS Pohlmann AR Silva PMRE Martins MA . Resveratrol-loaded lipid-core nanocapsules modulate acute lung inflammation and oxidative imbalance induced by LPS in mice. Pharmaceutics. (2021) 13:683. doi: 10.3390/pharmaceutics13050683 34068619 PMC8151102

[86] Adel IM ElMeligy MF Abdelrahim MEA Maged A Abdelkhalek AA Abdelmoteleb AMM . Design and characterization of spray-dried proliposomes for the pulmonary delivery of curcumin. Int J Nanomedicine. (2021) 16:2667–87. doi: 10.2147/IJN.S306831 33854314 PMC8039018

[87] Zhao S Jiang Q Yu L Liu Q Chen Z Zhan D . D-mannose-modified M2 macrophage membrane-derived decoy nanovesicles for synergistic immunotherapy of asthma. ACS Nano. (2026) 20:208–24. doi: 10.1021/acsnano.5c04996 41441854

[88] Beck C Ramanujam D Vaccarello P Widenmeyer F Feuerherd M Cheng C . Trimannose-coupled antimiR-21 for macrophage-targeted inhalation treatment of acute inflammatory lung damage. Nat Commun. (2023) 14:4564. doi: 10.1038/s41467-023-40185-1 37507393 PMC10382532

[89] Jin H Luo R Li J Zhao H Ouyang S Yao Y . Inhaled platelet vesicle-decoyed biomimetic nanoparticles attenuate inflammatory lung injury. Front Pharmacol. (2022) 13:1050224. doi: 10.3389/fphar.2022.1050224 36523494 PMC9745055

[90] Ouyang S Lu P Li J Jin H Wu W Luo R . Inhaled tea polyphenol-loaded nanoparticles coated with platelet membranes largely attenuate asthmatic inflammation. Respir Res. (2024) 25:311. doi: 10.1186/s12931-024-02947-3 39154188 PMC11330596

[91] Hayasaka N Yaita T Kuwaki T Honma S Honma K Kudo T . Optimization of dosing schedule of daily inhalant dexamethasone to minimize phase shifting of clock gene expression rhythm in the lungs of the asthma mouse model. Endocrinology. (2007) 148:3316–26. doi: 10.1210/en.2007-0010 17412811

[92] Teppan J Bärnthaler T Farzi A Durrington H Gioan-Tavernier G Platt H . The molecular circadian clock of eosinophils: a potential therapeutic target for asthma. Am J Physiol Cell Physiol. (2025) 328:C1394–408. doi: 10.1152/ajpcell.00149.2025 40131242 PMC7617784

[93] Joulia R Patti S Traves WJ Loewenthal L Yates L Walker SA . A single-cell spatial chart of the airway wall reveals proinflammatory cellular ecosystems and their interactions in health and asthma. Nat Immunol. (2025) 26:920–33. doi: 10.1038/s41590-025-02161-3 40399607 PMC12133579

[94] Yu X Li L Cai B Zhang W Liu Q Li N . Single-cell analysis reveals alterations in cellular composition and cell-cell communication associated with airway inflammation and remodeling in asthma. Respir Res. (2024) 25:76. doi: 10.1186/s12931-024-02706-4 38317239 PMC10845530

[95] Alladina J Smith NP Kooistra T Slowikowski K Kernin IJ Deguine J . A human model of asthma exacerbation reveals transcriptional programs and cell circuits specific to allergic asthma. Sci Immunol. (2023) 8:eabq6352. doi: 10.1126/sciimmunol.abq6352 37146132 PMC10440046

